# Identifying Predictive Bacterial Markers from Cervical Swab Microbiota on Pregnancy Outcome in Woman Undergoing Assisted Reproductive Technologies

**DOI:** 10.3390/jcm11030680

**Published:** 2022-01-28

**Authors:** Annacandida Villani, Andrea Fontana, Stefano Barone, Silvia de Stefani, Mariangela Primiterra, Massimiliano Copetti, Concetta Panebianco, Cristiana Parri, Natale Sciannamè, Pasqua Anna Quitadamo, Alessandra Tiezzi, Liliana Santana, Annamaria Maglione, Federica D’Amato, Francesco Perri, Simone Palini, Valerio Pazienza

**Affiliations:** 1 Gastoenterology Unit, Fondazione IRCCS “Casa Sollievo della Sofferenza” Hospital, Viale Padre Pio 4, 71013 San Giovanni Rotondo, Italy; a.villani@operapadrepio.it (A.V.); panebianco.c@gmail.com (C.P.); f.perri@operapadrepio.it (F.P.); 2 Unit of Biostatistic, Fondazione IRCCS “Casa Sollievo della Sofferenza” Hospital, Viale Padre Pio 4, 71013 San Giovanni Rotondo, Italy; a.fontana@operapadrepio.it (A.F.); m.copetti@operapadrepio.it (M.C.); 3 Unità Sanitaria Locale USL Toscana Nordovest, Assisted Reproductive Center Ospedale Versilia, 55041 Lido di Camaiore, Italy; barone.stefano@gmail.com (S.B.); cristiana.parri@uslnordovest.toscana.it (C.P.); 4 PMA Unit, Clinica Nuova Ricerca, Via Settembrini 17/h, 47923 Rimini, Italy; silviadestefani@ymail.com (S.d.S.); primiterra.pma@nuovaricerca.com (M.P.); tiezzi.pma@nuovaricerca.com (A.T.); santana.pma@nuovaricerca.com (L.S.); 5 Gynecology and Obstetrics Department, IRCCS “Casa Sollievo della Sofferenza”, 71013 San Giovanni Rotondo, Italy; n.scianname@operapadrepio.it (N.S.); a.maglione@operapadrepio.it (A.M.); 6 Neonatology Unit, Fondazione IRCCS “Casa Sollievo della Sofferenza”, 71013 San Giovanni Rotondo, Italy; pasquaq@tiscali.it; 7 PMA Unit, Villa Margherita Hospital, Viale di Villa Massimo, 48, 00161 Roma, Italy; damato.federica196@gmail.com; 8 Pathophysiology of Reproduction Unit, Ospedale “Cervesi” di Cattolica—AUSL Romagna, Via Ludwig Van Beethoven, 1, 47841 Cattolica, Italy; simonepalini@yahoo.it

**Keywords:** microbiota, assisted reproduction technology, pregnancy outcome, biomarkers

## Abstract

Background and aims: Failure of the embryo to implant causes about three-fourths of lost pregnancies. Female genital tract microbiota has been associated to Assisted Reproductive Technologies (ART) outcomes. The objective of this study was to analyze the microbiota of human cervical swab and to correlate these findings with the ART outcomes. Materials and Methods: In this study, 88 cervical swabs were collected from women undergoing ART cycles, with various causes of infertility, at the beginning of the ART protocols. After microbial DNA extraction, V3–V4 variable regions of the 16S rRNA gene were amplified and sequenced on the Illumina MiSeq platform. PEnalized LOgistic Regression Analysis (PELORA) was performed to identify clusters of bacterial populations with differential abundances between patients with unfavorable and favorable pregnancy outcome groups, respectively. Results: We identified a core of microorganisms at lower taxonomic levels that were predictive of women’s pregnancy outcomes. Statistically significant differences were identified at species levels with *Lactobacillus salivarius, Lactobacillus rhamnosus* among others. Moreover the abundance of *Lactobacillus crispatus* and *iners,* respectively increased and decreased in favorable group as compared to unfavorable group, resulted within the core of microorganisms associated to positive ART outcome. Although the predominance of lactobacilli is generally considered to be advantageous for ART outcome, we found that also the presence of Bifidobacterium (together with the other lactobacilli) was more abundant in the favorable group. Discussion: Cervix is colonized by microorganisms which can play a role in ART outcomes as seen by an overall decrease in embryo attachment rates and pregnancy rates in both fertile and infertile women. If confirmed in a larger cohort, the abundance of these bacteria can be useful not only as a marker of unfavorable pregnancy outcome but also they may open the way to new interventional strategies based on genital tract microbiota manipulation in order to increase the pregnancy rates in woman undergoing assisted reproductive technologies.

## 1. Introduction

Although around 95% of the trillion microorganisms constituting the microbiota reside within the gut, the remaining 5% are dislocated in other human districts including genital tracts. Vaginal microbiota is characterized by a lower bacterial diversity and high relative abundance of Lactobacillus species whose dominance is even more conspicuous during pregnancy [1]. Vaginal microbiota composition is strongly influenced by genetic, environmental, individual and lifestyle factors [2]. Recent evidence supports that the composition of the cervicovaginal microbiota plays a role in pregnancy outcome and it can be linked to adverse obstetric outcomes such as preterm birth, a leading cause of neonatal morbidity and mortality worldwide [3,4]. Moreover the microorganisms residing within the female reproductive tract have been associated with Assisted Reproductive Technologies (ART) outcomes [5]. One of the most important clinical challenges in this field is to improve the outcome of patients undergoing ART ever since the first live birth took place in 1978 [6]. Over the years, numerous studies have focused on investigating the importance of some factors related to ART failure, for instance sperm quality and female age. Beside the role of viral infections interfering with pregnancy outcome as previously published [7,8,9], over time, another factor has joined those mentioned above, such as the microbial composition of the female reproductive tract [10,11]. One of the pioneers in demonstrating the presence of endometrial microorganisms associated with ART outcomes was Moreno et al. in 2016 [12], giving life to a line of research aimed at improving the reproductive health of women with a focus on the cervicovaginal microbiota. The composition of cervicovaginal microbiota is characterized by high abundance of the Lactobacillus genus, some of which, such as *L. crispatus*, *L. gasseri* and *L. jensenii*, are able to introduce lactic acid and hydrogen peroxide (H_2_O_2_) in the female reproductive tract, inhibiting the growth of other bacteria and viruses [13]. Several studies have classified the cervicovaginal microbiota of reproductive-age women into six groups named Community State Types (CSTs), each of which is characterized by a predominant species: *Lactobacillus crispatus* (CST I), *Lactobacillus gasseri* (CST II), *Lactobacillus iners* (CST III), *Lactobacillus jensenii* (CST V) and CST IV-A and CST IV-B clusters. The last two groups are made up of a wide range of anaerobic and facultative bacteria, such as Gardnerella, Megasphera, Atopobium and Prevotella [3,14]. Particularly, CST IV-A differs from CST IV-B for the higher abundance of bacterial vaginosis-associated bacterium 1 (BVAB1) a species of bacteria associated with common vaginal disorders and belonging to the order of Clostridiales [15]. Up to date, only a limited number of studies regarding the relationship between female genital tract microbiota and pregnancy outcome are available. Cervical swab is the current method to obtain the biological matrix to analyze the microbiota of the cervix due to its minimally invasive impact. Given the anatomical structure of this district, one of the advantages of the cervical swab is the reduction of cross-contamination risk at sampling time [16]. Our study aimed to characterize the cervical swab microbiota of patients undergoing ART and to detect clusters of bacteria which were predictive (i.e., with differential abundance levels) of unfavorable and favorable pregnancy outcomes, respectively.

## 2. Materials and Methods

### 2.1. Study Population

Cervical swabs were collected immediately before carrying out the oocytes pickup and before external and internal disinfection from 90 women, diagnosed with different types of infertility, from November 2020 to May 2021, before undergoing assisted reproductive technology protocols at the outpatient division of Nuova Ricerca Hospital. All patients provided their signed informed consent. Ethical approval was obtained from the review boards of Human Ethics Committee of Nuova Ricerca Hospital under C.E. approval number 001\2020. Pregnancies were initially diagnosed by serum HCG and then confirmed as clinical pregnancies by ultrasound visualization of gestational sac with heartbeat. Out of 90 total patients, 2 of them were tested positive after embryo implantation but then had a miscarriage 8 weeks later, and for this reason they were excluded from the analysis. The remaining 88 patients were divided into two groups according to ART outcomes: 39 women resulted pregnant while the remaining 49 patients resulted negative. Exclusion criteria were: vaginal infection and antibiotic use 30 days before ART protocol, no previous pregnancy, previous history of pelvic inflammatory disease (PID), (PID), body mass index more than 30, age more than 40 and preimplantation test (PGT) positive for genetic diseases. Patients’ clinical data are described in Table 1.

### 2.2. Sample Collection and DNA Extraction

Cervical swab was collected from each study participant in a tube containing a DNA stabilization buffer (Copan Brescia Italy n. cat. 608C). After a centrifugation at 7500rpm for 10 min, total DNA was extracted using the QIAamp DNA blood and tissue Kit (Qiagen Milan Italy Cat. N. 69504) following the manufacturer’s instructions. At the end of the isolation protocol, DNA was checked for concentration and purity and stored at −80 °C until use.

### 2.3. Next-Generation Sequencing of Bacterial 16S rRNA Gene

In total, 5 ng of each DNA was utilized to amplify the V3-V4 region using KAPA HiFi HotStart ReadyMix (Roche Diagnostics, Milan, Italy, Cat N° 07958935001) and the following primers with Illumina adapters: forward primer: 5′-TCGTCGGCAGCGTCAGATGTGTATAAGAGACAGCCTACGGGNGGCWGCAG, reverse primer: 5′-GTCTCGTGGGCTCGGAGATGTGTATAAGAGACAGGACTACHVGGGTATCTAATCC, selected from Klindworth et al. [17]. Samples were barcoded with Nextera XT Index Kit (Illumina, Milan, Italy, Cat N° FC-131-1002). After pooling the libraries in equimolar concentrations, the libraries were subjected to 2 × 300 paired-end sequencing, using the MiSeq Reagent Kit v3 (600 cycle) (Illumina, Milan, Italy, Cat N ° MS-102-3003). FASTq files generated by MiSeq were processed using the 16S Metagenomics GAIA v.2.0 software as described in Fontana et al. [18]. Read pairs were quality-controlled (i.e., trimming, clipping and adapter removal) based on FastQC and BBDuk and mapped with BWA-MEM against the custom databases (based on NCBI), to obtain the taxonomic profile of each sample).

### 2.4. Statistical Analysis

Demographic and clinical characteristics of patients with unfavorable and favorable pregnancy outcomes were reported as mean ± standard deviation (SD), median along with interquartile range (i.e., first–third quartiles) and observed frequencies (and percentages) for continuous and categorical variables, respectively. For each continuous variable, the assumption of normality distribution was checked by means of quantile–quantile (Q-Q) plots and Shapiro–Wilks test. Comparisons between groups were performed by two-sample *t*-test and Chi-square test (or Fisher exact test as appropriate) for continuous and categorical variables, respectively. Stacked bar charts were used to show the vaginal microbiota composition (i.e., mean relative abundance %) at phylum, family, genus and species levels between patients with unfavorable and favorable pregnancy outcomes. To identify clusters of bacterial populations such that the linear combination of their abundances was differential between patients with unfavorable and favorable pregnancy outcomes, the PEnalized LOgistic Regression Analysis (PELORA) was performed [19]. This promising algorithm is mainly used to find predictive gene signatures from microarray data by using supervised grouping techniques. To this purpose, a standardized Z-score of each bacterium relative abundance (%) was computed as follows: as a first step, the abundance was logit transformed (i.e., computing the natural logarithm of the ratio between the relative abundance proportion and its complimentary) and, as a second step, the logit-transformed variable was standardized by subtracting its mean and dividing by its SD. When the relative abundance was exactly 0%, the logit transformation cannot be performed for that value and, to overcome this issue, such percentage was replaced by 0.001% for the computation of Z-score only. Using PELORA algorithm, multiple clusters of bacterial populations can be detected. Each cluster has the characteristic that its centroid (i.e., the mean of the Z-scores of all identified bacteria within the cluster) was significantly higher (or lower) in one of the two compared groups (i.e., patients with unfavorable and favorable pregnancy outcomes). Two different free parameters must be set by the user in the PELORA algorithm: the number of centroids and the penalty parameter (λ). The number of centroids was set to vary between one and two, because we were mainly interested to detect no more than two informative pathways for each scenario, whereas a number of different combinations of λ = (0, 1/32, 1/16, 1/8, 1/4, 1/2, 1) were evaluated, performing 200 bootstrap resamplings of the data and recording the overall misclassification rate. For each specific scenario, the penalty parameter that achieved the lowest median misclassification rate (across the boostrap samples) was chosen. Comparisons between Z-score means were assessed by two-sample *t*-test. Scatter plots (or box plots) of the Z-scores computed at cluster centroids as well as heatmaps of the relative bacteria abundance (%) identified by PELORA within each cluster were shown at phylum, family, genus and species levels. Two-sided *p* < 0.05 were considered to be statistically significant. All statistical analyses and plots were performed by the computing environment R (R Development Core Team 2008, version 4.1, packages: supclust, ggplot2, gridExtra).

## 3. Results

### 3.1. Sample Characteristics

Since it is known that bacteria may influence pregnancy outcome, the study participants undergoing ART were classified in two subgroups, according to whether they had a favorable or unfavorable pregnancy outcome at the end of the study. Clinical/pathological and demographic characteristics of these two subgroups of patients undergoing ART are summarized in Table 1. The two groups were homogeneous for all the examined characteristics except for Oligo-Astheno-Teratozoospermia (OAT) distribution (*p* = 0.002).

### 3.2. Comparison of Cervical Fluid Microbiota Composition between Patients with Favorable or Unfavorable ART Outcome

**Figure 1:**
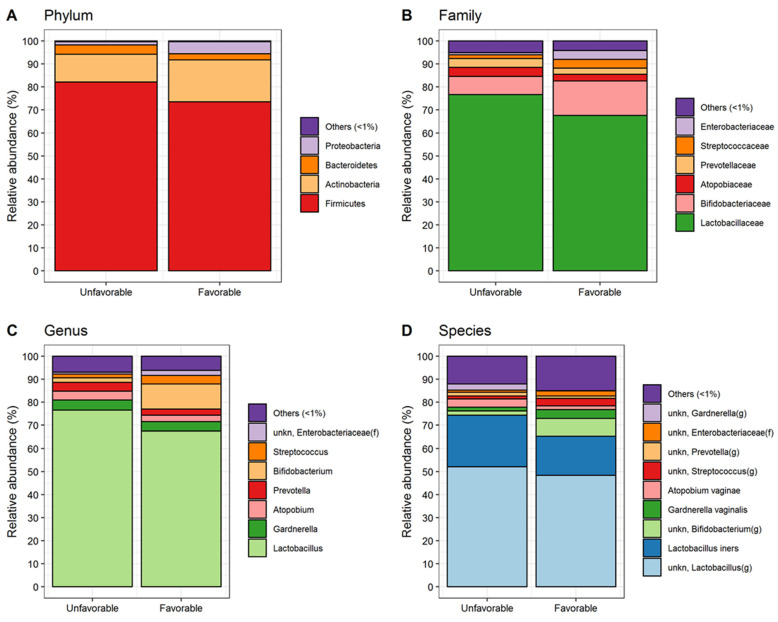
Cervical swab microbiota composition (i.e., mean relative abundance %) at phylum (**A**), family (**B**), genus (**C**) and species (**D**) levels in patients with unfavorable and favorable pregnancy outcome. All bacteria with mean relative abundance less than 1% are included in the “Others (<1%)” category.

In order to assess whether a different cervical fluid microbiota discriminates patients undergoing ART with a favorable or unfavorable outcome, its composition in the two cohorts of patients was analyzed by 16S rRNA gene sequencing. A total of 12,897,869 quality-filtered read pairs were obtained from 88 study participants with an average of 146,566 read pairs per sample (SD ± 69,272). Figure 1 reports cervical fluid bacterial communities at the phylum, family, genus and species level detected in 49 unfavorable and 39 favorable pregnancy outcome patients. As expected, Firmicutes was the most abundant phyla, accounting for about 82.2 and 73.5% of all bacteria without no significant changes between the unfavorable and favorable groups, respectively. The other most abundant phylum was constituted by the Actinobacteria. Consequently, Bidifobacteriaceae and Lactobacillaceae were the predominant families in both groups. At genus level, a significant increase of Bifidobacterium was detected within the favorable group while the unfavorable group was characterized by a significant increased presence of Atopobium. Worth of note, at species level, the increased relative abundance of *Lactobacillus iners* within the unfavorable group together with Atopobium vaginae which will be discussed below.

### 3.3. PELORA Algorithm Identified Bacterial Populations Associated to Favorable or Unfavorable Pregnancy Outcome

Based on the relative abundances generated by taxonomic analyses, the PELORA algorithm was performed to identify clusters of bacterial populations that best discriminate patients with favorable from those with unfavorable pregnancy outcome. The list of the bacteria detected by the algorithm within each cluster is reported in Table 2.

**Figure 2:**
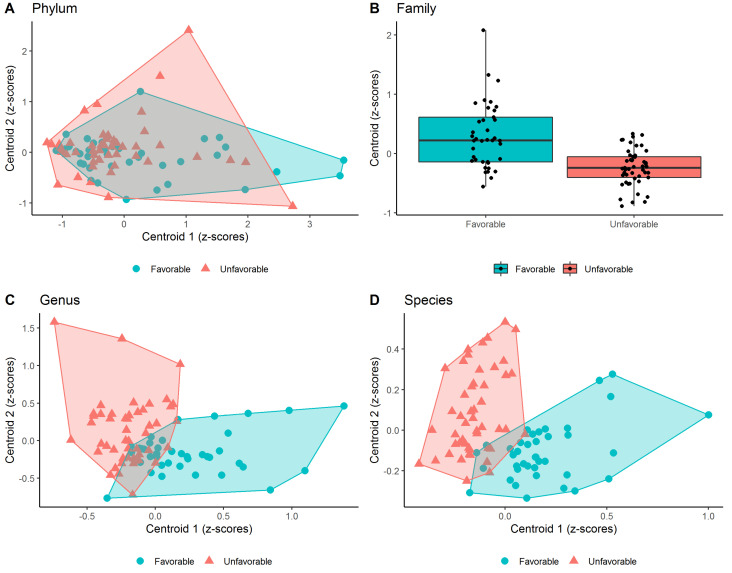
Scatter plots of the Z-scores computed within each cluster (i.e., centroid) detected by PEnalized LOgistic Regression Analysis at phylum (**A**), genus (**C**) and species (**D**). Each point represents the Z-scores pair computed at each individual and were filled with red and blue colors to denote patients with unfavorable and favorable pregnancy outcomes, respectively. Moreover, a polygon connecting the outermost data points is shown for each group. As a single cluster of bacteria population was detected at family level (**B**), the box plot was shown (instead of a scatter plot).

At phylum level, two clusters were detected but no significant differences were found both with respect to the cluster centroids and with respect to each bacterium included within each cluster. At the family level, only one cluster (which included 4 bacteria) was detected showing a significant increase in the abundance of unkn. Alphaproteobacteria (c), Yersiniaceae and Streptococcaceae as compared to the ones in the unfavorable group (*p* < 0.001; *p* < 0.007 and *p* < 0.036, respectively). Furthermore, the Z-scores of the cluster centroid were significantly different between the two groups (*p* < 0.001). At genus level, two clusters (which included 11 and 7 bacteria, respectively) were detected. The first one showed that a statistically significant increase in the abundance of unkn. Alphaproteobacteria (c) (*p* < 0.001), Yersinia (*p* < 0.009) and Streptococcus (*p* < 0.035). Bifidobacterium (*p* < 0.011), Enterobacter (*p* < 0.008), unkn. Sphingomonadaceae (f) (*p* < 0.002) and Micrococcus (*p* < 0.001) as well as the cluster’s centroid (*p* < 0.001) was found in patients with favorable pregnancy outcome with respect to the those with the unfavorable one. The second cluster showed that only a statistically significant increase in the abundance of the Peptoniphilus (*p* < 0.025) as well as the cluster’s centroid (*p* < 0.001) was found in patients with unfavorable pregnancy outcome with respect to the those with the favorable one. At the species level, two clusters (which included 22 and 20 bacteria, respectively) were detected. The first one showed that a statistically significant increase in the abundance unkn. Alphaproteobacteria (c) (*p* < 0.001), unkn. Serratia (g) (*p* < 0.001), Lactobacillus psittaci (*p* < 0.045), unkn. Bifidobacterium (g) (*p* < 0.019), Streptococcus anginosus (*p* < 0.004), Yersinia pseudotuberculosis (*p* < 0.029), Lactobacillus casei (*p* < 0.010), unkn. Sphingomonadaceae (f) (*p* < 0.002) and unkn. Micrococcus (g) (*p* < 0.001) as well as the cluster’s centroid (*p* < 0.001) was found in patients with favorable pregnancy outcome with respect to the those with the unfavorable one. Instead, the second cluster showed that a statistically significant increase in the abundance of the of unkn. Firmicutes (p) (*p* < 0.033), Anaerococcus prevotii (*p* < 0.023). Peptoniphilus lacrimalis (*p* < 0.041) and Peptoniphilus timonensis (*p* < 0.041) as well as the cluster’s centroid (*p* < 0.001) was found in patients with unfavorable pregnancy outcome with respect to the those with the favorable one. It is of note that the latter two bacteria were found to be completely absent among patients with the unfavorable outcome. The distribution of Z-scores computed at clusters centroids was graphically represented in Figure 2 at different taxa levels, showing that two clusters, composed by the linear combination of specific microorganisms residing within the cervical fluid, were able to greatly discriminate (except at phylum level) patients with unfavorable and favorable pregnancy outcomes, respectively.

**Figure 3:**
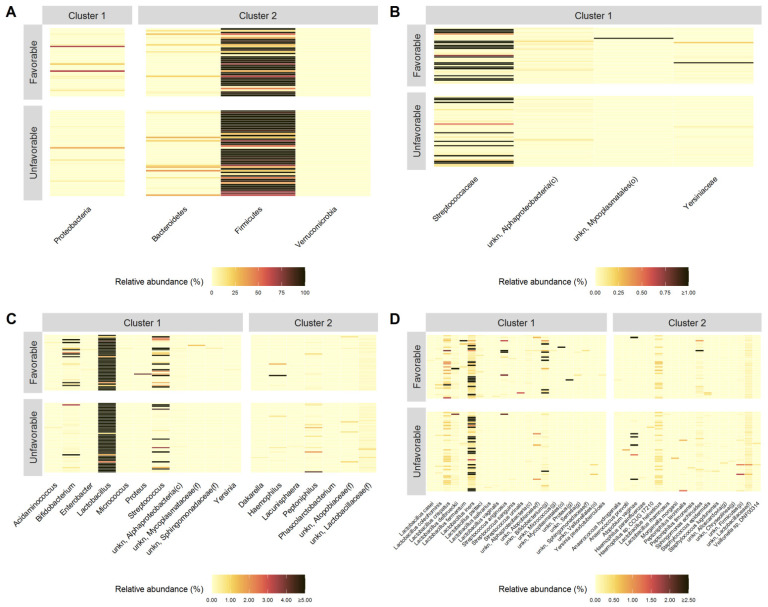
Heatmaps of relative abundance (%) of bacterial populations identified (into different clusters) by the PEnalized LOgistic Regression Analysis at phylum (**A**) family (**B**), genus (**C**) and species (**D**) levels, grouped by patients with unfavorable and favorable pregnancy outcomes, respectively.

Specifically, patients with an unfavorable outcome are characterized by lower Z-scores from cluster 1 and higher Z-scores from cluster 2, whereas, on the contrary, those with a favorable outcome are characterized by higher Z-scores from cluster 1 and lower Z-scores from cluster 2. Because of the presence of a single cluster in the family level, it was quite clear that the centroid’s Z-scores detected within patients with unfavorable outcome were significantly lower than the ones with the favorable one. Moreover, Heatmaps reported in Figure 3 show the relative abundance of each of the microorganisms detected within each cluster at the phylum (A), family (B), genus (C) and species (D) level for each recruited subject in the unfavorable and favorable groups, respectively.

## 4. Discussion

Vaginal microbiota has been investigated in several studies highlighting its importance in maintaining a healthy female reproductive system. Differences in the composition of bacteria that populate the vaginal tract can be the reason for vaginal infections or disfunctions [20]. Although no statistical significance was observed for tubal pathology, of note is the fact that it is present in 6.8% of all subjects while only one (2%) was present in the unfavorable group and five (12.8%) in the favorable group (Table 1), underlining the importance of a core microbiota involved in the pregnancy rate. The known importance of microbiota residing within the reproductive system and in reproductive health led us to analyze whether the microbiota composition may influence the outcome of ART cycles. For this reason, previous studies analyzed how the prevalence of some bacteria or others can influence ART outcomes such as the implantation rate or abortion rate [21]. Moreover, in the last few years the presence of bacteria in the highest parts of the female reproductive system has been detected, such as the uterus and ovaries [5]. In this upper part of reproductive system, the composition of microbiota is different from the vaginal one. In particular, the uterine microbiota is characterized by a lower diversity in terms of number of bacteria species as compared to the vaginal microbiota [22]. Differently from the vaginal microorganisms, the upper reproductive tract was considered sterile and was little investigated until several years ago. Different studies then proved that all components of the upper reproductive tract, i.e., the uterus, fallopian tubes and ovaries, are populated by different species of bacteria [21,22,23]. The population of bacteria of the upper reproductive tract has proved to be less abundant than the vaginal one but contains a richer variety of different species. Lactobacillus are still the most abundant species, but less so than in the vaginal tract. Bacteria that populates the uterine microbiota has been proposed to be responsible for protecting the endometrium from infection and modulating its function [24]. Uterine microbiota can be analyzed to understand how it influences embryo implantation. Nevertheless, samples of endometrium tissue are really hard to obtain and invasive for patients. For these reasons, studies that analyzes uterine microbiota are still low in number. This study had the purpose to analyze the spectrum of species that populate the uterine cervix in patients that undergoing ART. The use of cervical swabs, in fact, could give a result more similar to the uterine cervix than the vaginal one. It was already reported that the presence of a poor Lactobacilli-dominant microbiota has been correlated with a higher probability of failure in in vitro fecundation (IVF) treatment [12]. Previous studies use different methods to analyze microbiota composition and different techniques to obtain samples. For these reasons, more studies has still to be carried out to understand how the microbiota of the reproductive female tract could influence ART outcomes. The discovery of an “ideal” core of microorganisms which are able to increase the implantation chances could lay the groundwork to use therapies that modulate bacterial composition. We found a core of microorganisms, listed in Table 2, whose abundance or scarcity is associated with a favorable or unfavorable ART outcome. For instance, the increased abundance of *Atopobium vaginae* within the unfavorable ART outcome can be explained by the fact that this microorganism is associated to a bacterial vaginosis [25], reducing the rate of pregnancy success [26,27]. Moreover, our data showed that *Lactobacillus crispatus* and *Lactobacillus iners* increased and decreased, respectively, in the favorable group as compared to unfavorable group. As reported in a previous study, their abundance over a certain limit is also important for the ART outcome [10]. Lactobacilli are fundamental as they lower the vaginal pH through the production of lactic acid, generating an unfavorable habitat for many pathogens [28]. Based on this rationale, the indication that colonizing the reproductive tract microbiota with different species of Lactobacillus to achieve a “healthy” profile through the administration of H_2_O_2_-producing *L. crispatus* could enhance the success rate of ART outcome emerged [29]. However, although the predominance of lactobacilli is generally considered to be advantageous for ART outcome [10], we found that also the presence of Bifidobacterium (together with the other lactobacilli) was more abundant in the favorable group. Indeed, it was suggested that bifidobacteria contribute to a healthy vaginal microbiota [25] and associated to a lower risk of preterm birth [26]. Our data suggests that cervical swab microbiota profiles could be useful not only to detect markers of unfavorable pregnancy outcome, if confirmed in larger cohorts, but also in paving the way for new interventional strategies based on genital tract microbiota manipulation in order to increase the pregnancy rates in woman undergoing assisted reproductive technologies.

## Figures and Tables

**Table 1:** Demographic and clinical patients’ characteristics (overall and according to the pregnancy outcome).

Variable	Category	All Subjects (*N* = 88)	Unfavorable (*N* = 49)	Favorable (*N* = 39)	*p*-Value
Age (years)	Mean ± SD	35.1 ± 3.0	35.3 ± 3.4	35.0 ± 2.6	0.659 *
	Median (IQR)	36 (33–37)	36 (32–38)	35 (33–37)	
	Range (min–max)	24–40	29–39	24–40	
BMI (Kg/m^2^)	Mean ± SD	22.1 ± 3.2	22.2 ± 3.1	22.0 ± 3.4	0.770 *
	Median (IQR)	21.5 (19.9–23.1)	21.6 (20–22.6)	21.3 (19.9–23.3)	
	Range (min–max)	16.1–32.8	16.1–32.5	17.6–32.8	
Infertility—N(%)	1 = Male infertility	20 (22.7)	13 (26.5)	7 (17.9)	0.627 ^#^
	2 = Idiopathic	13 (14.8)	8 (16.3)	5 (12.8)	
	3 = Low ovarian reserve	18 (20.5)	10 (20.4)	8 (20.5)	
	1 + 3 = Male and Low ovarian reserve	3 (3.4)	1 (2.0)	2 (5.1)	
	4 = Ovulatory endocrine	10 (11.4)	6 (12.2)	4 (10.3)	
	5 = Endometriosis	3 (3.4)	2 (4.1)	1 (2.6)	
	6 = Multifactorials	15 (17.0)	8 (16.3)	7 (17.9)	
	7 = Tubal inferitility	6 (6.8)	1 (2.0)	5 (12.8)	
OAT—N(%)	1 = Normal	29 (33.0)	10 (20.4)	19 (48.7)	
	2 = Moderate	50 (56.8)	36 (73.5)	14 (35.9)	0.002 ^#^
	3 = Severe	9 (10.2)	3 (6.1)	6 (15.4)	
FSH—N(%)	1 = Meropur	17 (19.3)	10 (20.4)	7 (17.9)	0.518 ^#^
	2 = Pergoveris	27 (30.7)	13 (26.5)	14 (35.9)	
	3 = Bemfola	43 (48.9)	26 (53.1)	17 (43.6)	
	4 = Meropur + Ovaleap	1 (1.1)	0 (0.0)	1 (2.6)	
Diet—N(%)	1 = Mediterranean	74 (84.1)	42 (85.7)	32 (82.1)	0.862 ^§^
	2 = Vegetarian/Vegan	14 (15.9)	7 (14.3)	7 (17.9)	
Physical activity—N(%)	1 = Low-intensity	16 (18.2)	7 (14.3)	9 (23.1)	0.675 ^#^
	2 = Moderate-intensity	64 (72.7)	37 (75.5)	27 (69.2)	
	3 = High-intensity	8 (9.1)	5 (10.2)	3 (7.7)	
Smoking habits—N(%)	1 = Smoker	24 (27.3)	12 (24.5)	12 (30.8)	0.677 ^§^
	2 = Non-smoker	64 (72.7)	37 (75.5)	27 (69.2)	
Drink habits—N(%)	1 = Drinker	20 (22.7)	12 (24.5)	8 (20.5)	0.835 ^§^
	2 = Non-drinker	52 (59.1)	29 (59.2)	23 (59.0)	
	3 = Occasional-drinker	16 (18.2)	8 (16.3)	8 (20.5)	
Sexual activity—N(%)	1 = <1 a week	33 (37.5)	18 (36.7)	15 (38.5)	0.777 ^§^
	2 = 1–2 a week	41 (46.6)	22 (44.9)	19 (48.7)	
	3 = >2 a week	14 (15.9)	9 (18.4)	5 (12.8)	

Missing values are excluded, and only valid percentages are reported. * *p*-value from two-sample test; ^§^ *p*-value from Chi-Square test; ^#^ *p*-value from Fisher exact test. Abbreviations: SD—standard deviation; OAT—Oligo-Astheno-Teratozoospermia.

**Table 2:** Results from PEnalized LOgistic Regression Analysis (PELORA). The PELORA algorithm identified clusters of bacterial populations such that the linear combination of their abundances (Z-scores) is differential between patients with unfavorable and favorable pregnancy outcomes, respectively.

Taxa Level	Cluster Number	Selected Bacteria (within Each Cluster)	Quantity	Statistics	Unfavorable (*N* = 49)	Favorable (*N* = 39)	*p*-Value ^#^
Phylum	1	Proteobacteria * (Cluster Centroid)	Relative abundance (%)	Mean ± SD	1.399 ± 4.952	5.140 ± 15.088	-
				Median (IQR)	0.216 (0.146–0.364)	0.267 (0.138–1.238)	
			Z-score°	Mean ± SD	−0.175 ± 0.804	0.220 ± 1.176	0.065
	2	Verrucomicrobia	Relative abundance (%)	Mean ± SD	0.012 ± 0.074	0.001 ± 0.002	-
				Median (IQR)	0.000 (0.000–0.000)	0.000 (0.000–0.000)	
			Z-score°	Mean ± SD	0.105 ± 1.251	−0.132 ± 0.529	0.273
		Bacteroidetes	Relative abundance (%)	Mean ± SD	4.091 ± 9.730	2.709 ± 6.412	-
				Median (IQR)	0.102 (0.022–0.384)	0.062 (0.021–0.283)	
			Z-score°	Mean ± SD	0.068 ± 0.997	−0.086 ± 1.010	0.477
		Firmicutes	Relative abundance (%)	Mean ± SD	82.180 ± 27.847	73.507 ± 35.960	-
				Median (IQR)	96.456 (74.673–99.494)	96.301 (40.668–99.508)	
			Z-score°	Mean ± SD	0.084 ± 0.921	−0.106 ± 1.095	0.378
		Cluster centroid	Z-score (means)	Mean ± SD	0.086 ± 0.545	−0.108 ± 0.379	0.063
Family	1	unkn, Alphaproteobacteria(c)	Relative abundance (%)	Mean ± SD	0.013 ± 0.020	0.031 ± 0.036	-
				Median (IQR)	0.006 (0.003–0.015)	0.020 (0.010–0.038)	
			Z-score°	Mean ± SD	−0.373 ± 0.915	0.468 ± 0.910	<0.001
		Yersiniaceae	Relative abundance (%)	Mean ± SD	0.005 ± 0.013	0.039 ± 0.162	-
				Median (IQR)	0.001 (0.000–0.003)	0.003 (0.000–0.010)	
			Z-score°	Mean ± SD	−0.252 ± 0.808	0.317 ± 1.131	0.007
		Streptococcaceae	Relative abundance (%)	Mean ± SD	1.646 ± 5.198	3.808 ± 10.311	-
				Median (IQR)	0.028 (0.016–0.145)	0.061 (0.024–2.155)	
			Z-score°	Mean ± SD	−0.199 ± 0.896	0.250 ± 1.077	0.036
		unkn, Mycoplasmatales (o)	Relative abundance (%)	Mean ± SD	0.000 ± 0.001	0.101 ± 0.633	-
				Median (IQR)	0.000 (0.000–0.000)	0.000 (0.000–0.000)	
			Z-score°	Mean ± SD	−0.118 ± 0.258	0.148 ± 1.471	0.219
		Cluster centroid	Z-score (means)	Mean ± SD	−0.235 ± 0.307	0.296 ± 0.545	<0.001
Genus	1	unkn, Alphaproteobacteria (c)	Relative abundance (%)	Mean ± SD	0.013 ± 0.019	0.031 ± 0.036	-
				Median (IQR)	0.006 (0.003–0.015)	0.020 (0.010–0.038)	
			Z-score°	Mean ± SD	−0.373 ± 0.914	0.469 ± 0.910	<0.001
		Yersinia	Relative abundance (%)	Mean ± SD	0.000 ± 0.000	0.006 ± 0.028	-
				Median (IQR)	0.000 (0.000–0.000)	0.000 (0.000–0.001)	
			Z-score°	Mean ± SD	−0.246 ± 0.370	0.309 ± 1.393	0.009
		Bifidobacterium	Relative abundance (%)	Mean ± SD	1.940 ± 12.454	10.856 ± 26.752	-
				Median (IQR)	0.065 (0.030–0.112)	0.076 (0.038–0.646)	
			Z-score°	Mean ± SD	−0.239 ± 0.669	0.300 ± 1.248	0.011
		unkn, Mycoplasmataceae (f)	Relative abundance (%)	Mean ± SD	0.000 ± 0.001	0.032 ± 0.198	-
				Median (IQR)	0.000 (0.000–0.000)	0.000 (0.000–0.000)	
			Z-score°	Mean ± SD	−0.175 ± 0.385	0.219 ± 1.419	0.066
		Proteus	Relative abundance (%)	Mean ± SD	0.000 ± 0.000	0.104 ± 0.650	-
				Median (IQR)	0.000 (0.000–0.000)	0.000 (0.000–0.000)	
			Z-score°	Mean ± SD	−0.148 ± 0.300	0.186 ± 1.453	0.120
		Enterobacter	Relative abundance (%)	Mean ± SD	0.004 ± 0.012	0.020 ± 0.066	-
				Median (IQR)	0.001 (0.000–0.003)	0.002 (0.000–0.006)	
			Z-score°	Mean ± SD	−0.249 ± 0.826	0.313 ± 1.117	0.008
		unkn, Sphingomonadaceae (f)	Relative abundance (%)	Mean ± SD	0.004 ± 0.006	0.015 ± 0.058	-
				Median (IQR)	0.002 (0.001–0.004)	0.004 (0.003–0.007)	
			Z-score°	Mean ± SD	−0.295 ± 0.876	0.371 ± 1.033	0.002
		Acidaminococcus	Relative abundance (%)	Mean ± SD	0.009 ± 0.059	0.010 ± 0.043	-
				Median (IQR)	0.000 (0.000–0.000)	0.000 (0.000–0.000)	
			Z-score°	Mean ± SD	−0.061 ± 0.927	0.077 ± 1.092	0.521
		Streptococcus	Relative abundance (%)	Mean ± SD	1.645 ± 5.195	3.807 ± 10.310	-
				Median (IQR)	0.028 (0.016–0.144)	0.059 (0.023–2.155)	
			Z-score°	Mean ± SD	−0.200 ± 0.899	0.251 ± 1.074	0.035
		Lactobacillus	Relative abundance (%)	Mean ± SD	76.525 ± 32.162	67.472 ± 38.152	-
				Median (IQR)	92.876 (67.735–98.344)	91.819 (35.048–98.003)	
			Z-score°	Mean ± SD	0.092 ± 0.931	−0.115 ± 1.082	0.338
		Micrococcus	Relative abundance (%)	Mean ± SD	0.001 ± 0.002	0.007 ± 0.028	-
				Median (IQR)	0.000 (0.000–0.001)	0.000 (0.000–0.003)	
			Z-score°	Mean ± SD	−0.296 ± 0.620	0.372 ± 1.245	0.001
		Cluster centroid	Z-score (means)	Mean ± SD	−0.199 ± 0.193	0.250 ± 0.381	<0.001
	2	Peptoniphilus	Relative abundance (%)	Mean ± SD	0.240 ± 0.701	0.053 ± 0.154	-
				Median (IQR)	0.012 (0.006–0.066)	0.008 (0.000–0.028)	
			Z-score°	Mean ± SD	0.211 ± 1.037	−0.266 ± 0.896	0.025
		unkn, Lactobacillaceae (f)	Relative abundance (%)	Mean ± SD	0.173 ± 0.147	0.130 ± 0.046	-
				Median (IQR)	0.145 (0.119–0.172)	0.133 (0.102–0.162)	
			Z-score°	Mean ± SD	0.176 ± 1.019	−0.222 ± 0.941	0.063
		Lacunisphaera	Relative abundance (%)	Mean ± SD	0.003 ± 0.022	0.000 ± 0.000	-
				Median (IQR)	0.000 (0.000–0.000)	0.000 (0.000–0.000)	
			Z-score°	Mean ± SD	0.113 ± 1.335	−0.142 ± 0.011	0.236
		Dakarella	Relative abundance (%)	Mean ± SD	0.007 ± 0.042	0.000 ± 0.000	-
				Median (IQR)	0.000 (0.000–0.000)	0.000 (0.000–0.000)	
			Z-score°	Mean ± SD	0.152 ± 1.312	−0.191 ± 0.216	0.110
		Haemophilus	Relative abundance (%)	Mean ± SD	0.038 ± 0.139	0.169 ± 0.809	-
				Median (IQR)	0.000 (0.000–0.003)	0.000 (0.000–0.001)	
			Z-score°	Mean ± SD	0.057 ± 0.959	−0.071 ± 1.058	0.554
		unkn, Atopobiaceae (f)	Relative abundance (%)	Mean ± SD	0.062 ± 0.222	0.027 ± 0.116	-
				Median (IQR)	0.003 (0.002–0.007)	0.002 (0.001–0.006)	
			Z-score°	Mean ± SD	0.104 ± 1.055	−0.131 ± 0.923	0.277
		Phascolarctobacterium	Relative abundance (%)	Mean ± SD	0.003 ± 0.020	0.000 ± 0.001	-
				Median (IQR)	0.000 (0.000–0.000)	0.000 (0.000–0.000)	
			Z-score°	Mean ± SD	0.095 ± 1.300	−0.119 ± 0.359	0.322
		Cluster centroid	Z-score (means)	Mean ± SD	0.130 ± 0.437	−0.163 ± 0.271	<0.001
Species	1	unkn, Alphaproteobacteria (c)	Relative abundance (%)	Mean ± SD	0.013 ± 0.019	0.031 ± 0.036	-
				Median (IQR)	0.006 (0.003–0.015)	0.020 (0.010–0.038)	
			Z-score°	Mean ± SD	−0.373 ± 0.914	0.469 ± 0.910	<0.001
		unkn, Serratia (g)	Relative abundance (%)	Mean ± SD	0.003 ± 0.006	0.014 ± 0.047	-
				Median (IQR)	0.001 (0.000–0.001)	0.002 (0.000–0.008)	
			Z-score°	Mean ± SD	−0.299 ± 0.775	0.376 ± 1.127	0.001
		*Lactobacillus psittaci*	Relative abundance (%)	Mean ± SD	0.009 ± 0.012	0.036 ± 0.071	-
				Median (IQR)	0.006 (0.003–0.010)	0.009 (0.003–0.020)	
			Z-score°	Mean ± SD	−0.190 ± 0.729	0.239 ± 1.230	0.045
		unkn, Bifidobacterium (g)	Relative abundance (%)	Mean ± SD	1.793 ± 12.073	7.734 ± 22.655	-
				Median (IQR)	0.037 (0.016–0.063)	0.046 (0.026–0.339)	
			Z-score°	Mean ± SD	−0.222 ± 0.699	0.279 ± 1.236	0.019
		*Streptococcus sanguinis*	Relative abundance (%)	Mean ± SD	0.005 ± 0.016	0.008 ± 0.027	-
				Median (IQR)	0.000 (0.000–0.002)	0.000 (0.000–0.005)	
			Z-score°	Mean ± SD	−0.133 ± 0.960	0.167 ± 1.036	0.163
		unkn, Mycoplasmatales (o)	Relative abundance (%)	Mean ± SD	0.000 ± 0.001	0.101 ± 0.633	-
				Median (IQR)	0.000 (0.000–0.000)	0.000 (0.000–0.000)	
			Z-score°	Mean ± SD	−0.118 ± 0.258	0.148 ± 1.471	0.219
		*Streptococcus anginosus*	Relative abundance (%)	Mean ± SD	0.045 ± 0.294	0.314 ± 0.990	-
				Median (IQR)	0.001 (0.000–0.002)	0.002 (0.000–0.023)	
			Z-score°	Mean ± SD	−0.271 ± 0.684	0.340 ± 1.218	0.004
		*Lactobacillus crispatus*	Relative abundance (%)	Mean ± SD	0.221 ± 0.226	0.452 ± 0.724	-
				Median (IQR)	0.089 (0.016–0.482)	0.171 (0.026–0.583)	
			Z-score°	Mean ± SD	−0.103 ± 0.949	0.130 ± 1.059	0.280
		*Yersinia pseudotuberculosis*	Relative abundance (%)	Mean ± SD	0.000 ± 0.000	0.005 ± 0.026	-
				Median (IQR)	0.000 (0.000–0.000)	0.000 (0.000–0.000)	
			Z-score°	Mean ± SD	−0.207 ± 0.289	0.260 ± 1.435	0.029
		*Lactobacillus fermentum*	Relative abundance (%)	Mean ± SD	0.007 ± 0.035	0.086 ± 0.398	-
				Median (IQR)	0.000 (0.000–0.001)	0.000 (0.000–0.001)	
			Z-score°	Mean ± SD	−0.142 ± 0.757	0.178 ± 1.228	0.137
		*Lactobacillus coleohominis*	Relative abundance (%)	Mean ± SD	0.014 ± 0.053	0.032 ± 0.098	-
				Median (IQR)	0.000 (0.000–0.000)	0.000 (0.000–0.001)	
			Z-score°	Mean ± SD	−0.131 ± 0.863	0.164 ± 1.139	0.170
		*Streptococcus urinalis*	Relative abundance (%)	Mean ± SD	0.000 ± 0.000	0.039 ± 0.243	-
				Median (IQR)	0.000 (0.000–0.000)	0.000 (0.000–0.000)	
			Z-score°	Mean ± SD	−0.150 ± 0.157	0.189 ± 1.481	0.114
		*Lactobacillus casei*	Relative abundance (%)	Mean ± SD	0.000 ± 0.001	0.003 ± 0.017	-
				Median (IQR)	0.000 (0.000–0.001)	0.000 (0.000–0.000)	
			Z-score°	Mean ± SD	−0.242 ± 0.667	0.305 ± 1.248	0.010
		unkn, Proteus (g)	Relative abundance (%)	Mean ± SD	0.000 ± 0.000	0.068 ± 0.423	-
				Median (IQR)	0.000 (0.000–0.000)	0.000 (0.000–0.000)	
			Z-score°	Mean ± SD	−0.142 ± 0.266	0.178 ± 1.463	0.137
		unkn, Sphingomonadaceae (f)	Relative abundance (%)	Mean ± SD	0.004 ± 0.006	0.015 ± 0.058	-
				Median (IQR)	0.002 (0.001–0.004)	0.004 (0.003–0.007)	
			Z-score°	Mean ± SD	−0.295 ± 0.876	0.371 ± 1.033	0.002
		*Lactobacillus vaginalis*	Relative abundance (%)	Mean ± SD	0.001 ± 0.008	0.005 ± 0.030	-
				Median (IQR)	0.000 (0.000–0.000)	0.000 (0.000–0.000)	
			Z-score°	Mean ± SD	−0.094 ± 0.813	0.118 ± 1.195	0.327
		unkn, Micrococcus (g)	Relative abundance (%)	Mean ± SD	0.001 ± 0.002	0.006 ± 0.028	-
				Median (IQR)	0.000 (0.000–0.001)	0.000 (0.000–0.002)	
			Z-score°	Mean ± SD	−0.307 ± 0.614	0.386 ± 1.241	0.001
		*Lactobacillus iners*	Relative abundance (%)	Mean ± SD	22.349 ± 36.633	16.920 ± 32.134	-
				Median (IQR)	0.380 (0.214–34.653)	0.428 (0.284–1.786)	
			Z-score°	Mean ± SD	0.068 ± 1.067	−0.086 ± 0.916	0.475
		*Lactobacillus delbrueckii*	Relative abundance (%)	Mean ± SD	0.093 ± 0.274	0.531 ± 2.978	-
				Median (IQR)	0.037 (0.011–0.094)	0.036 (0.013–0.103)	
			Z-score°	Mean ± SD	−0.057 ± 0.995	0.072 ± 1.014	0.552
		unkn, Atopobiaceae (f)	Relative abundance (%)	Mean ± SD	0.062 ± 0.222	0.027 ± 0.116	-
				Median (IQR)	0.003 (0.002–0.007)	0.002 (0.001–0.006)	
			Z-score°	Mean ± SD	0.104 ± 1.055	−0.131 ± 0.923	0.277
		*Lactobacillus salivarius*	Relative abundance (%)	Mean ± SD	0.004 ± 0.022	0.000 ± 0.001	-
				Median (IQR)	0.000 (0.000–0.000)	0.000 (0.000–0.000)	
			Z-score°	Mean ± SD	−0.043 ± 1.317	0.054 ± 0.303	0.655
		unkn, Veillonellales (o)	Relative abundance (%)	Mean ± SD	0.021 ± 0.098	0.001 ± 0.003	-
				Median (IQR)	0.000 (0.000–0.001)	0.000 (0.000–0.000)	
			Z-score°	Mean ± SD	0.052 ± 1.270	−0.065 ± 0.496	0.591
		Cluster centroid	Z-score (means)	Mean ± SD	−0.150 ± 0.106	0.188 ± 0.222	<0.001
	2	unkn, Lactobacillaceae (f)	Relative abundance (%)	Mean ± SD	0.173 ± 0.147	0.130 ± 0.046	-
				Median (IQR)	0.145 (0.119–0.172)	0.133 (0.102–0.162)	
			Z-score°	Mean ± SD	0.176 ± 1.019	−0.222 ± 0.941	0.063
		*Anaerococcus prevotii*	Relative abundance (%)	Mean ± SD	0.018 ± 0.109	0.000 ± 0.001	-
				Median (IQR)	0.000 (0.000–0.000)	0.000 (0.000–0.000)	
			Z-score°	Mean ± SD	0.215 ± 1.275	−0.270 ± 0.320	0.023
		*Staphylococcus epidermidis*	Relative abundance (%)	Mean ± SD	0.010 ± 0.027	0.021 ± 0.093	-
				Median (IQR)	0.000 (0.000–0.000)	0.000 (0.000–0.000)	
			Z-score°	Mean ± SD	0.110 ± 1.023	−0.139 ± 0.965	0.249
		*Atopobium vaginae*	Relative abundance (%)	Mean ± SD	3.620 ± 13.759	1.647 ± 10.286	-
				Median (IQR)	0.000 (0.000–0.000)	0.000 (0.000–0.000)	
			Z-score°	Mean ± SD	0.145 ± 1.173	−0.183 ± 0.700	0.127
		unkn, Firmicutes (p)	Relative abundance (%)	Mean ± SD	0.080 ± 0.282	0.013 ± 0.013	-
				Median (IQR)	0.011 (0.007–0.024)	0.009 (0.006–0.013)	
			Z-score°	Mean ± SD	0.201 ± 1.141	−0.253 ± 0.725	0.033
		unkn, Chryseolinea (g)	Relative abundance (%)	Mean ± SD	0.003 ± 0.018	0.000 ± 0.001	-
				Median (IQR)	0.000 (0.000–0.000)	0.000 (0.000–0.000)	
			Z-score°	Mean ± SD	0.054 ± 1.291	−0.068 ± 0.420	0.573
		*Haemophilus parainfluenzae*	Relative abundance (%)	Mean ± SD	0.003 ± 0.019	0.000 ± 0.001	-
				Median (IQR)	0.000 (0.000–0.000)	0.000 (0.000–0.000)	
			Z-score°	Mean ± SD	0.072 ± 1.300	−0.090 ± 0.375	0.454
		*Anaerococcus hydrogenalis*	Relative abundance (%)	Mean ± SD	0.002 ± 0.006	0.004 ± 0.020	-
				Median (IQR)	0.000 (0.000–0.000)	0.000 (0.000–0.000)	
			Z-score°	Mean ± SD	0.081 ± 1.035	−0.101 ± 0.957	0.399
		*Lactobacillus salivarius*	Relative abundance (%)	Mean ± SD	0.004 ± 0.022	0.000 ± 0.001	-
				Median (IQR)	0.000 (0.000–0.000)	0.000 (0.000–0.000)	
			Z-score°	Mean ± SD	−0.043 ± 1.317	0.054 ± 0.303	0.655
		*Peptoniphilus lacrimalis*	Relative abundance (%)	Mean ± SD	0.043 ± 0.210	Absent	-
				Median (IQR)	0.000 (0.000–0.000)		
			Z-score°	Mean ± SD	0.173 ± 1.321	Absent	0.041 ^§^
		*Staphylococcus lugdunensis*	Relative abundance (%)	Mean ± SD	0.006 ± 0.040	0.000 ± 0.003	-
				Median (IQR)	0.000 (0.000–0.000)	0.000 (0.000–0.000)	
			Z-score°	Mean ± SD	0.044 ± 1.205	−0.055 ± 0.669	0.646
		*Haemophilus* sp., CCUG 17210	Relative abundance (%)	Mean ± SD	0.009 ± 0.061	Absent	-
				Median (IQR)	0.000 (0.000–0.000)		
			Z-score°	Mean ± SD	0.085 ± 1.340	Absent	0.372 ^§^
		*Lactobacillus rhamnosus*	Relative abundance (%)	Mean ± SD	0.003 ± 0.018	0.000 ± 0.002	-
				Median (IQR)	0.000 (0.000–0.000)	0.000 (0.000–0.000)	
			Z-score°	Mean ± SD	−0.004 ± 1.203	0.005 ± 0.678	0.966
		*Peptoniphilus timonensis*	Relative abundance (%)	Mean ± SD	0.007 (0.047)	Absent	-
				Median (IQR)	0.000 (0.000–0.000)		
			Z-score°	Mean ± SD	0.065 ± 1.343	Absent	0.041 ^§^
		*Morganella morganii*	Relative abundance (%)	Mean ± SD	0.014 ± 0.098	0.000 ± 0.002	-
				Median (IQR)	0.000 (0.000–0.000)	0.000 (0.000–0.000)	
			Z-score°	Mean ± SD	0.022 ± 1.244	−0.027 ± 0.578	0.822
		*Veillonella* sp., DNF00314	Relative abundance (%)	Mean ± SD	0.012 ± 0.060	Absent	-
				Median (IQR)	0.000 (0.000–0.000)		
			Z-score°	Mean ± SD	0.121 ± 1.334	Absent	0.204 ^§^
		*Sphingomonas echinoides*	Relative abundance (%)	Mean ± SD	0.059 ± 0.070	0.206 ± 0.770	-
				Median (IQR)	0.043 (0.000–0.079)	0.040 (0.000–0.135)	
			Z-score°	Mean ± SD	0.052 ± 0.870	−0.065 ± 1.151	0.587
		*Lactobacillus iners*	Relative abundance (%)	Mean ± SD	22.349 ± 36.633	16.920 ± 32.134	-
				Median (IQR)	0.380 (0.214–34.653)	0.428 (0.284–1.786)	
			Z-score°	Mean ± SD	0.068 ± 1.067	−0.086 ± 0.916	0.475
		*Lactobacillus helveticus*	Relative abundance (%)	Mean ± SD	0.176 ± 0.180	0.183 ± 0.217	-
				Median (IQR)	0.047 (0.019–0.384)	0.049 (0.017–0.407)	
			Z-score°	Mean ± SD	0.040 ± 0.964	−0.051 ± 1.054	0.673
		unkn, Alloscardovia (g)	Relative abundance (%)	Mean ± SD	0.008 ± 0.039	0.003 ± 0.008	-
				Median (IQR)	0.000 (0.000–0.000)	0.000 (0.000–0.000)	
			Z-score°	Mean ± SD	0.029 ± 1.072	−0.037 ± 0.914	0.760
		Cluster centroid	Z-score (means)	Mean ± SD	0.085 ± 0.207	−0.107 ± 0.140	<0.001

Abbreviations: IQR—interquartile range (i.e., first–third quartiles); SD—standard deviation; Absent—all values are 0%. * This is a one-element cluster: the Proteobacteria represents a cluster in itself and therefore its Z-score mean corresponds to the centroid Z-score mean. Standardized Z-score: as a first step, the relative abundance (%) of each bacterium was logit transformed (so that values can theoretically range from negative to positive infinity) and, as a second step, the Z-score was computed by standardizing the transformed variable (i.e., taking the variable values, subtracting its mean and dividing by its SD). The centroid is calculated as the average of Z-scores for all those variables selected within each cluster. ^#^ All *p*-values were derived from the parametric two-sample *t*-test on Z-scores with the exception of those marked as “^§^” which instead were derived from Mann–Whitney U test. The latter was performed in presence of no variance in one of the two groups (i.e., when the group has all values equals to 0%—denoted as “Absent”).

## Data Availability

Data regarding this study are available upon reasonable request.
